# Neighborhood environmental attributes and walking mobility decline: A longitudinal ecological study of mid-to-older aged Australian adults

**DOI:** 10.1371/journal.pone.0252017

**Published:** 2021-06-03

**Authors:** Takemi Sugiyama, Masaaki Sugiyama, Suzanne Mavoa, Anthony Barnett, Md. Kamruzzaman, Gavin Turrell

**Affiliations:** 1 Centre for Urban Transitions, Swinburne University of Technology, Hawthorn, VIC, Australia; 2 Mary MacKillop Institute for Health Research, Australian Catholic University, Melbourne, VIC, Australia; 3 Baker Heart and Diabetes Institute, Melbourne, VIC, Australia; 4 Graduate School of Human Life Science, Osaka City University, Osaka, Japan; 5 Melbourne School of Population & Global Health, University of Melbourne, Melbourne, VIC, Australia; 6 Monash Urban Planning and Design, Monash University, Caulfield East, VIC, Australia; 7 Centre for Research and Action in Public Health, Health Research Institute, University of Canberra, ACT, Australia; Peking University Shenzhen Graduate School, CHINA

## Abstract

**Objectives:**

Cross-sectional studies have found some built environmental attributes to be associated with residents’ lower levels of mobility (functional capacity to walk outside the home). However, less is known about what environmental attributes are related to mobility decline. This longitudinal study examined area-level associations of specific environmental attributes with mid-to-older aged adults’ changes in walking mobility.

**Methods:**

Data collected from 4,088 adults (aged 46–71 years at baseline) who participated in a cohort study in Brisbane, Australia were used. The outcome was the change in self-reported mobility score (SF-36) from 2013 to 2016, which were aggregated at the neighborhood (N = 156) and suburb (N = 99) levels, due to the known lack of sensitivity in SF-36 subscales to individual changes. Linear regression analysis examined associations of mobility change with seven environmental attributes measured at baseline (residential density, intersection density, land use mix, density of walking/bike paths, park density, bus stop density, density of social incivilities), adjusting for confounding variables.

**Results:**

Participants on average reported 4% of mobility decline during the 3-year study period. It was found that greater land use diversity was consistently associated with less decline in walking mobility, while greater density of social incivilities was associated with more decline in walking mobility. The latter finding was significant only at the neighborhood level. No consistent associations were observed for residential density, intersection density, density of walking/bike paths, park density, and bus stop density.

**Discussion:**

Our findings suggest that mid-to-older aged adults who live in areas with lower land use diversity and more social incivilities may be at risk of developing mobility limitations. Recommended policies to slow residents’ mobility decline and to achieve aging in place include improving these environmental attributes where needed and advising older adults to relocate to safer, mixed-use neighborhoods.

## Introduction

Aging in place, where older adults continue to live independently in their homes within the local community, is a key policy objective in the context of population ageing and increasing healthy life expectancy [1]. Aging in place is an important strategy to reduce the high cost of providing residential care for an increasing number of older adults requiring care [2] and to meet the aspirations of many older adults to stay in a familiar local environment [3].

Walking mobility, defined as the functional ability to walk outside the home, is fundamental to achieving aging in place, as this capacity is needed to carry out important activities of daily living, such as accessing local shops and services, engaging in recreational activities, and participating in social events [4, 5]. However, walking mobility declines gradually as people age, due in large part to decreasing muscle strength, increasing pain, illness, and sensory impairment [6]. Although there are individual-level interventions to improve walking mobility, they tend to be small in scale and poor in post-intervention sustainability [7]. Long-term, wide-reaching prevention strategies to combat mobility decline are required to promote aging in place [1]. Given that age-related declines in mobility begin in middle age and continue throughout the later stages of life [8], it is important to develop population-level strategies to delay or slow declines in walking mobility not just among older adults but also among middle-aged adults.

The environments where older people live are relevant to their walking mobility [9, 10]. Local environments facilitate or hinder daily physical activity, such as walking [11–14], which is known to influence middle-to-older aged adults’ mobility overtime [15, 16]. World Health Organization’s *Age-friendly Cities and Communities* project is an example of an environmental strategy to foster healthy and active aging [17]. Although increasing research has examined how built environmental attributes are related to older adults’ mobility and physical function, a recent review has shown that about two thirds of the studies on this topic are cross-sectional in design [18].

There are several longitudinal studies on the built environment and mobility. It can be postulated that mobility is easier to maintain in more “walkable” areas, where local shops and services are more accessible due to higher population density, better street connectivity, and more diverse land use. However, studies examining longitudinal relationships between walkability attributes and walking mobility are limited and have produced mixed findings. For instance, an American study reported that middle-aged African Americans living in areas with lower street connectivity were more likely to develop lower-body functional limitations than those living in areas that were higher in street connectivity [19]. However, another American study showed no impact of street connectivity on older women’s change in lower extremity physical function [20]. In addition, a study of Dutch older adults found that the number of local destinations was not associated with a repeated measure of limitations in carrying out daily activities [21].

There are also a few longitudinal studies examining the role of perceived safety (such as vandalism, litter, and crime) in older adults’ mobility. Two studies found greater perceived safety to be associated with improvement in mobility [22] and with reduced odds of developing functional limitations [23], while one study reported the onset of functional disability to be associated with perceived neighborhood problems but not with perceived neighborhood crime [24]. However, no longitudinal study has examined the relationship of an objective measure of neighborhood crime and mobility decline.

Understanding which environmental attributes are protective against mobility decline is an important step in developing population-level strategies to promote aging in place. This study examined longitudinal associations of objectively derived walkability attributes and safety with decline in walking mobility among mid-to-older aged adults in Brisbane, Australia.

## Methods

### Study design: Units of analysis

Research on the built environment and functional decline has been conducted at the individual level. However, the instrument to measure the outcome in this study was found not sensitive enough to assess individual change over time [25]. To overcome this methodological issue, which is discussed in more detail in the section of Outcome Measure below, we conducted an ecological analysis, in which the outcome measure was aggregated at the neighborhood and suburb levels. Neighborhoods corresponded to a Census Collection District (CCD) that is a small geographical unit, comprising about 250 households [26]. Suburbs, which consist of multiple CCDs, typically contain retail areas and surrounding residential areas (both in inner urban and peri-urban areas). They are an approximation of established local areas, which were used as a component of a postal address [26]. Characteristics of neighborhoods and suburbs are shown in the Results section.

### Data source

This longitudinal study used data from the HABITAT study. Details about HABITAT’s sampling design have been published elsewhere [27]. Briefly, a multi-stage probability sampling design was used to select a stratified random sample of 200 CCDs in Brisbane (S1 Fig). From each CCD, a random sample of people aged 40–65 years was selected in 2007. The initial sample included 11,035 adults who returned the questionnaire with useable data (response rate: 69%). This study used data collected in the 3rd (2013, baseline for this study) and 4th (2016, follow-up) waves of data collection. The number of participants who provided data in both waves was 4,784 (retention rate = 80%). The median follow-up duration was 3 years and 2 months. Their age at baseline ranged from 46 to 71 years. The Human Research Ethics Committee of the Queensland University of Technology (Ref. no. 3967H) gave ethics approval for the HABITAT study.

### Outcome measure

The outcome of the study was change in walking mobility from 2013 to 2016. Three items related to walking and two items related to stair climbing in the Physical Function 10 (PF10), a subscale of the Short Form 36 Health Survey (SF-36), were used for this purpose. These items asked participants to indicate how much their health limits them in walking 100 m, walking half a kilometer, walking 1 km, climbing one flight of stairs, and climbing several flights of stairs. We used these items as walking and climbing stairs are necessary to carry out daily shopping and errands outside the home, and they are directly relevant to neighborhood environments. In addition, self-reported difficulty in walking and climbing a stair was shown to be closely associated with gait speed measures in mid-to-older aged adults [28]. The response options were: 1 for “Yes, limited a lot”; 2 for “Yes, limited a little”; and 3 for “No, not limited at all”. Following the SF-36 scoring protocol, the mean score was converted into 0 to 100, with 100 being without any limitation for all the behaviors and 0 being limited a lot for all the items.

It has been shown that sensitivity to individual changes in SF-36 subscales is very low, even among those who underwent an orthopedic surgery [25]. The study of orthopedic surgery patients found that there was a large amount of measurement error at the individual level, but it detected meaningful changes in group scores following the surgery [25]. This measurement issue may be more serious in the current observational study, where changes observed are age-related and gradual. Since the ability to detect change in walking mobility score is essential for this study, we employed ecological design, in which we aggregated participant’s mobility score at area levels. This design should not impair the objective of this study, as we sought to understand which environmental attributes are protective against area-level mobility decline. Such area-level findings would be informative, since what matters to environmental initiatives, which are implemented for areas, is to enhance health of the areas (not necessarily each person in the areas).

### Exposure measures

The following environmental attributes calculated for the third wave (2013) at the neighborhood and suburb levels were used: residential density; 4-way intersection density; land use mix; density of walking/bike paths; density of parks; density of bus stops; and density of social incivilities. These were chosen, as they were attributes often found to be associated with walking by older adults [11]. For residential density, residential cadasters were derived by joining cadaster and land use data sourced from the Brisbane City Council and selecting cadasters with residential land use codes. Residential density was calculated by dividing the number of residential cadasters included in or intersecting each area (neighborhood or suburb) by its area size. Intersection density was calculated by dividing the number of 4-way intersections within an area, which were derived from street network data (Pitney Bowes Pro 2013; PSMA 2016, accessed via AURIN), by the area size. To calculate land use mix, area size for five land use categories (commercial, industrial, leisure/recreation, residential, and other) within each area were obtained from cadaster data. An entropy measure of a neighborhood or suburb was calculated from an equation described elsewhere [29]. Walking/bike path data were available from a site of the Brisbane City Council (www.data.brisbane.qld.gov.au). The density was calculated by dividing the length of paths intersecting each area by its area size. For park density, it was calculated by dividing the total area of parks within each area by the area size. Density of bus stops was calculated as the number of bus stops within each area, sourced from the Brisbane City Council, divided by its size. Density of train stations was not examined in this study, as there were only a few neighborhoods that had a train station. Density of social incivilities was calculated as the total number of incivilities in the area divided by its size. Social incivilities include drug offences, prostitution offences, trespassing and vagrancy, and good order offences [30]. Crime data were supplied by the Queensland Police.

### Covariates

Participants reported their age, gender, education, work status, household composition, and household income. For each area unit, the mean age, the mean proportion of women, those with high school qualification or less, those working, those living as a couple, and those with higher income (over $93,600 per annum) at baseline were calculated at each area level and adjusted for in analyses. We also adjusted for area size of neighborhoods or suburbs, as their size may affect the outcome through unmeasured confounders (e.g., social norms and support for active living). Participants’ reasons for choosing their current address were also used as a covariate, as such reasons and associated attitudes may affect their daily behaviors and the trajectory of functional status. Participants reported the importance of various reasons for choosing their current address in a format ranging from 1 (“not at all important”) to 5 (“very important”). We used the mean of the following seven items related to ease of walking (one item), access to local destinations (five items: shops, public transport, greenery, open space, recreational facilities) and safety from crime (one item), measured at 2007 or after relocation between 2007 and 2013 (α = 0.83). The composite score, which indicates participant’s preference for walk-friendly and safe areas, was aggregated at the neighborhood or suburb levels. Study areas were ranked according to the level of socio-economic status, using the Socio-Economic Indexes for Areas [31].

### Sample size and statistical analyses

The initial sample size (N = 4,784) was reduced to 4,278 after excluding those who earlier in the study had relocated outside of Brisbane (n = 506), where there were no geographic data. For the outcome measure, participants who missed three or more items (n = 57) and those who scored 0 at baseline (n = 133) were excluded, leaving 4,088 participants. The latter exclusion was made because mobility of those who already had difficulty walking 100 m and climbing one flight of stairs at baseline are unlikely to be influenced by neighborhood environments. Participants who moved outside their neighborhoods (n = 202) or outside their suburbs (n = 181) during the study period were also excluded. The number of participants retained was 3,886 for neighborhood-level analyses and 3,907 for suburb-level analyses. These participants resided in 513 neighborhoods and in 155 suburbs. Since many participants relocated between 2007 and 2013, there are neighborhoods and suburbs where only a few participants resided. Area units with less than 10 participants were removed from analyses, resulting in 156 neighborhoods and 99 suburbs. As this is an arbitrary cut-off, we also conducted analyses using areas with 5 or more participants (195 neighborhoods, 113 suburbs) and those with 15 or more participants (107 neighborhoods, 88 suburbs).

We ran linear regression analyses using the change in walking mobility score (follow-up–baseline) as the outcome at the level of neighborhood or suburb. At each level, two models were fitted. Single variable model (SVM) examined each environmental attribute individually (not adjusting for each other), while a multi-variable model (MVM) examined all environmental attributes simultaneously (adjusting for each other). All models adjusted for individual-level socio-demographic factors, area size, and preference for walk-friendly and safe areas (mean calculated for each area unit) and area-level socio-economic status. Analyses were conducted using Stata 16 (StataCorp, College Station, TX). Statistical significance was set at p < 0.05.

## Results

Table 1 shows the characteristics of the neighborhoods and suburbs included in analyses. Suburbs were about five times larger in geographic size and included twice more participants than neighborhoods in the study area. Overall, the mean walking mobility score decreased about 4% (from the low 90s at baseline to the high 80s at follow-up) during the 3-year study period. The values for environmental attributes were similar at the neighborhood and suburb levels, except for land use mix, where the values for suburbs were larger than those for neighborhoods. This is because suburbs are larger and tend to include more different land uses.

**Table 1:** Characteristics of neighborhoods and suburbs included in analyses.

	Neighborhoods (N = 156)	Suburbs (N = 99)
Mean size (SD), ha	103 (620)	531 (562)
Median number of participants [1Q, 3Q]	18 [13, 25]	31 [19, 49]
Mean age (SD)	57.9 (2.2)	57.9 (1.9)
Mean % of women (SD)	57.5 (11.8)	57.3 (9.5)
Mean % with high school or less (SD)	33.4 (15.1)	33.7 (14.4)
Mean % working (SD)	65.0 (13.3)	64.8 (9.7)
Mean % living as a couple (SD)	74.2 (14.5)	72.9 (13.8)
Mean % with income ≥ $93,600^a^ (SD)	39.9 (17.2)	40.8 (15.8)
Mean preference for walk-friendly and safe area (SD)	3.17 (0.30)	3.12 (0.25)
Mean SES percentile score (SD)	56.4 (26.9)	54.8 (24.2)
Mean walking mobility score (SD)		
At baseline	92.8 (4.0)	92.7 (3.7)
At follow up	89.2 (5.5)	88.8 (5.6)
Change (follow-up–baseline)	-3.6 (3.8)	-3.8 (3.6)
Mean environmental attributes		
Residential density (SD), count/ha	18.6 (15.3)	18.9 (11.3)
Intersection density (SD), count/ha	0.11 (0.12)	0.10 (0.06)
Land use mix (SD)	0.37 (0.23)	0.61 (0.14)
Density of walking/bike paths (SD), m/ha	23.3 (28.9)	21.8 (16.2)
Density of parks (SD), ha/ha	0.08 (0.12)	0.09 (0.07)
Density of bus stops (SD), count/ha	0.14 (0.12)	0.12 (0.07)
Density of social incivilities (SD), count/ha	0.21 (0.18)	0.19 (0.12)

^a^ Gross annual income in Australian dollars.

Table 2 shows Pearson’s correlation coefficients of environmental attributes at the neighborhood level (lower left half) and at the suburb level (upper right half). Correlation coefficients were generally larger at the suburb level, where some coefficients were 0.7 and over, than at the neighborhood level, where the largest coefficient was around 0.5. Table 3 shows regression coefficients indicating the association of each environmental attribute with walking mobility change at the neighborhood and suburb levels. The risk of multicollinearity was considered low in MVMs, as the variance inflation factor was all below 2 at the neighborhood level and below 5 at the suburb level. Negative coefficients can be interpreted as showing that a 1-SD increment in environmental attribute is associated with more decline in walking mobility score over the study period. At the neighborhood level, it was found that greater land use diversity was associated with less decline in walking mobility, while greater density of social incivilities was associated with more decline in walking mobility score in SVM. In MVM, the association of land use mix and density of social incivilities remained significant. Higher density of bus stops was marginally associated with more decline in walking mobility in SVM, but the association became non-significant in MVM. At the suburb level analyses, greater land use mix was associated with less decline in walking mobility in both SVM and MVM. Higher residential density was associated with more decline in walking mobility in MVM. Density of social incivilities, which was significant at the neighborhood level, was not associated with the outcome at the suburb level. Densities of street intersections, walking/bike paths, and parks were not related to walking mobility change in any models. Area-level socio-economic status was consistently associated with walking mobility change. In MVM, 1-SD increment in socio-economic status (higher) was associated with 1.07 less decline in mobility (95%CI: 0.19, 1.96, p = 0.018) at the neighborhood level and with 1.50 less decline (95%CI: 0.48, 2.52, p = 0.004) at the suburb level.

**Table 2:** Pearson’s correlation coefficients between environmental attributes.

	1	2	3	4	5	6	7
1. Residential density	−	0.70***	0.29**	0.74***	-0.04	0.57***	0.67***
2. Intersection density	0.16*	−	0.07	0.46***	-0.11	0.70***	0.70***
3. Land use mix	-0.05	-0.22**	−	0.09	0.24*	-0.03	-0.03
4. Density of walking/bike paths	0.33***	0.04	0.01	−	0.05	0.49***	0.59***
5. Density of parks	-0.07	-0.27***	0.53***	0.02	−	-0.15	-0.20*
6. Density of bus stops	0.06	0.37***	-0.23**	0.16*	-0.27***	−	0.57***
7. Density of social incivilities	0.42***	0.48***	-0.15	0.19*	-0.23**	0.30***	−

* p < 0.05

** p < 0.01

*** p < 0.001.

Coefficients shown in the lower left half are calculated at the neighborhood level (N = 156), while those shown in the upper right half are calculated at the suburb level (N = 99).

**Table 3:** Ecological associations of walking mobility score change with environmental attributes.

	Neighborhood level (N = 156)		Suburb level (N = 99)	
	SVMs	MVM	SVMs	MVM
Residential density	-0.20 (-0.81, 0.41)	0.01 (-0.65, 0.68)	-0.67 (-1.44, 0.09)†	-1.47 (-2.76, -0.17)*
Intersection density	-0.43 (-1.05, 0.18)	0.21 (-0.50, 0.91)	-0.40 (-1.16, 0.36)	0.36 (-0.82, 1.55)
Land use mix	0.71 (0.12, 1.31)*	0.68 (0.00, 1.37)*	0.90 (0.18, 1.63)*	1.43 (0.64, 2.23)***
Density of walking/bike paths	0.15 (-0.45, 0.76)	0.36 (-0.27, 1.00)	-0.50 (-1.28, 0.29)	0.16 (-0.89, 1.20)
Density of parks	0.32 (-0.27, 0.91)	-0.24 (-0.92, 0.44)	-0.31 (-1.01, 0.39)	-0.50 (-1.19, 0.20)
Density of bus stops	-0.58 (-1.18, 0.03)†	-0.35 (-1.00, 0.30)	-0.64 (-1.43, 0.15)	-0.45 (-1.42, 0.53)
Density of social incivilities	-0.91 (-1.50, -0.33)**	-0.96 (-1.68, -0.24)**	-0.37 (-1.15, 0.41)	0.31 (-0.74, 1.35)

† p < 0.1

* p < 0.05

** p < 0.01

*** p < 0.001.

Regression coefficients (corresponding to a 1-SD increment in each exposure measure) and 95% CI are shown.

SVMs (single variable models) examined each environmental attribute separately (not adjusted for each other). MVM (multi-variable model) examined all environmental attributes simultaneously (adjusted for each other).

All models adjusted for mean age, the mean proportion of women, those with high school qualification or less, those working, those living as a couple, and those with a higher income (over $93,600 per annum), area size, mean preference for walk-friendly and safe area, and area-level socio-economic status.

S1 Table shows the results of neighborhood-level analyses where different cut-off values for neighborhood selection were applied. Significant associations consistent with Table 3 were found for land use mix (except for MVM for neighborhoods with 5+ participants) and density of social incivilities (only in models with neighborhoods with 5+ participants). Density of walking/bike paths and that of parks were associated with less decline in walking mobility in one model but not in the other models. S2 Table shows the results of suburb-level analyses where different cut-off values for suburb selection were used. Again, land use mix was found related to less walking mobility decline in most models. But, social incivility density was associated with the outcome only in one model (SVM for suburbs with 5+ participants). Similar to Table 3, higher residential density was associated with more decline in walking mobility (SVM for suburbs with 5+ participants, MVM for suburbs with 15+ participants). Higher intersection density was also associated with greater decline in one model.

## Discussion

This longitudinal ecological study examined associations of environmental attributes with walking mobility change over 3 years among mid-to-older adults in Brisbane, Australia. Analyses employed different spatial units (neighborhood and suburb), different modelling (single variable and multi-variable models), and different cut-off values for area selection. Consistent evidence was found for land use mix and social incivilities. Greater land use diversity both at the neighborhood and suburb levels was found protective against walking mobility decline in this study sample. One-SD increment in land use mix was related to 0.7 to 1.4 less decline in walking mobility. Given that the mean mobility decline among all participants was 3.6 to 3.8 during the study period, it can be argued that neighborhoods or suburbs with diverse land use can provide a sizable benefit to residents’ mobility. Density of social incivilities was found relevant at the neighborhood level but not at the suburb level. A greater occurrence of various unlawful behaviors (drug, prostitution, trespassing, vagrancy, and good order offences) in close vicinity appears to speed up mobility decline among mid-to-older aged residents. However, the presence of such offences in a larger area (suburb) was not related to mobility decline. These findings were observed from analyses adjusting for area-level socioeconomic status, suggesting that the impact of social incivilities on walking mobility is independent of deprivation.

It can be argued that daily physical activity such as walking can be a pathway that explains the associations found: greater land use and better safety, which are known to be associated with older adults’ physical activity [11], can contribute to maintenance of walking mobility over time [15, 16]. However, the study did not find consistent associations for other environmental attributes that are also known to be related to physical activity, such as the density of dwellings, intersections, walking/bike paths, and parks. It should be noted that some associations of residential density and intersection density with mobility decline were found in the unexpected direction. It is not clear at this stage why land use mix and safety, but not other environmental attributes, were related to residents’ mobility decline. A literature review found several categories of risk factors of early functional decline: medical status, body composition, physical and mental status (including cognition), social participation (including motivation), demographics, and relationships with healthcare providers [32]. It is possible that local areas with more diverse land uses and fewer social incivilities encourage social participation, which may lead to mobility maintenance [33]. Further research is needed to better understand mechanisms through which local environments contribute to residents’ mobility maintenance.

Several regression models that differed in area units, modelling approach, and area selection were fitted. The differences between findings from SVM (single variable model) and MVM (multi-variable model) are worth discussing. SVM examined each environmental attribute separately, while MVM examined all of them simultaneously. In the latter, a regression coefficient of a particular attribute is obtained while holding other attributes constant. This assumption is not in line with the reality, where many environmental attributes are correlated with each other (particularly at the suburb level) as shown in Table 2. However, contrasting findings from SVM and MVM can be useful in some cases. For example, higher bus stop density at the neighborhood level was associated (marginally) with greater mobility decline in SVM, but the association became not significant in MVM (Table 3). It is possible that bus stop density was related to the outcome partly because it was correlated negatively with land use mix and positively with social incivility density, and adjusting for these variables may have attenuated the association. However, regression coefficients were magnified in MVM in some models (residential density and land use mix at the suburb level). Higher correlation between environmental attributes at the suburb level may have played a role in larger differences in regression coefficients between SVM and MVM.

Two previous longitudinal studies examined associations of street connectivity with functional decline. One found higher street connectivity to be protective against lower-body functional decline [19], while the other reported non-significant associations between them [20]. This study was consistent with the latter. It is notable that street connectivity was negatively correlated with land use mix (not correlated at the suburb level) and positively correlated with density of social incivilities. Since these two attributes were consistently associated with the outcome (land use mix positively, social incivilities negatively), such spatial relationships, which may be distinctive characteristics in the study area, may have contributed to non-significant associations for street connectivity. Regarding safety, the findings of the current study are in line with previous studies, which found poorer perceived safety (including perceived neighborhood problems) to be associated with a higher likelihood of developing functional limitations [22–24]. It is possible to argue that there is strong evidence of personal safety issues in the neighborhood having detrimental impact on residents’ physical function overtime.

A limitation of this study is its ecological design. We used an aggregated measure of walking mobility at an area level, due to the sensitivity issue identified in an experimental study [25]. The estimates produced in the analyses may be biased in depicting individual-level relationships. In addition, our study is subject to the modifiable area unit problem, where results can vary depending on the spatial unit used [34], and the uncertain geographic context problem, which is the challenge of identifying the area unit most relevant to the behavior or outcome under investigation [35]. Inability to use individual-level data of and a lack of knowledge on the spatial contexts relevant to mobility change mean that these challenges were difficult to address. However, we used two different area units with additional supplementary analyses (different criteria for area selection), which produced largely consistent findings. The approach focusing on areas may have some advantages over the one focusing on individuals, as identifying area-level environmental attributes that may be modified to address community’s health issues is key to inform the development of urban design initiatives [36, 37]. Our findings, higher levels of land use mix associated with less decline of walking mobility at an area level, would justify recommending diverse land use, as they suggest that such initiatives may benefit health of the area where they are implemented (if not individuals in the area). This further highlights the importance of selecting a proper spatial unit of analysis in examining contextual effects, as environmental strategies to influence outcomes of interest are implemented at different scales [38]. The outcome of the study was self-reported difficulty in walking and climbing stairs. Although they are part of a validated instrument (SF-36) to measure physical function, future research needs to corroborate the findings using physical function test scores. Finally, data were collected in Brisbane, Australia. The findings may not be generalizable to other localities. Strengths of the study include the use of a range of objectively assessed environmental attributes calculated at the neighborhood and suburb levels, including density of social incivilities. The study also accounted for possible confounding factors including residents’ preference for walk-friendly and safe area in choosing their residence.

## Conclusion

We found that mid-to-older aged adults who live in areas with lower land use diversity and more social incivilities are likely to experience more decline in walking mobility over three years, compared to those living in areas with higher land use diversity and fewer social incivilities. Our findings also suggest that the presence of social incivilities matters only in immediate surroundings, while land use diversity is relevant both at the neighborhood and suburb levels. Given that residential density and intersection density were not associated with mobility decline, our findings suggest that even low-density suburban areas can be protective against mid-to-older aged residents’ mobility decline, if they have mixed land use and have fewer safety concerns. However, it is important to replicate these findings in different contexts. It is also of interest to examine how changes in the built environments can influence mobility. Australian cities are experiencing population increase, which may not happen in a homogeneous manner [39]. Areas where population density increases may provide more retail opportunities, which may have beneficial effects on residents’ mobility change. Our findings inform urban planning and public health policies and initiatives to achieve aging in place. A relevant policy recommendation is to advise mid-to-older aged adults where to relocate. A considerable proportion of adults in retirement age relocate with a view to downsize their residence [40]. Those who move to safer, mixed-use neighborhoods may have a better chance of maintaining their mobility. Further research can investigate how measures of accessibility at local and regional scales, which are known to affect residents’ travel behaviors [41], are associated with mobility decline to inform policies related to older adults’ relocation and aging in place. It is also important that future research corroborates the findings of this study using objective measures of lower-body function and to identify behavioral mechanisms explaining the associations observed.

## Supporting information

S1 Fig (TIF)

S1 Table Ecological associations of walking mobility score change with environmental attributes at the neighborhood level. (DOCX)

S2 Table Ecological associations of walking mobility score change with environmental attributes at the suburb level. (DOCX)

S1 Data (DTA)

S2 Data (DTA)
