# Supplementary figures and images for: Neighborhood environmental attributes and walking mobility decline: A longitudinal ecological study of mid-to-older aged Australian adults
Source: PLoS One. 2021 Jun 3;16(6):e0252017. doi: 10.1371/journal.pone.0252017 (PMC8174704; doi:10.1371/journal.pone.0252017)

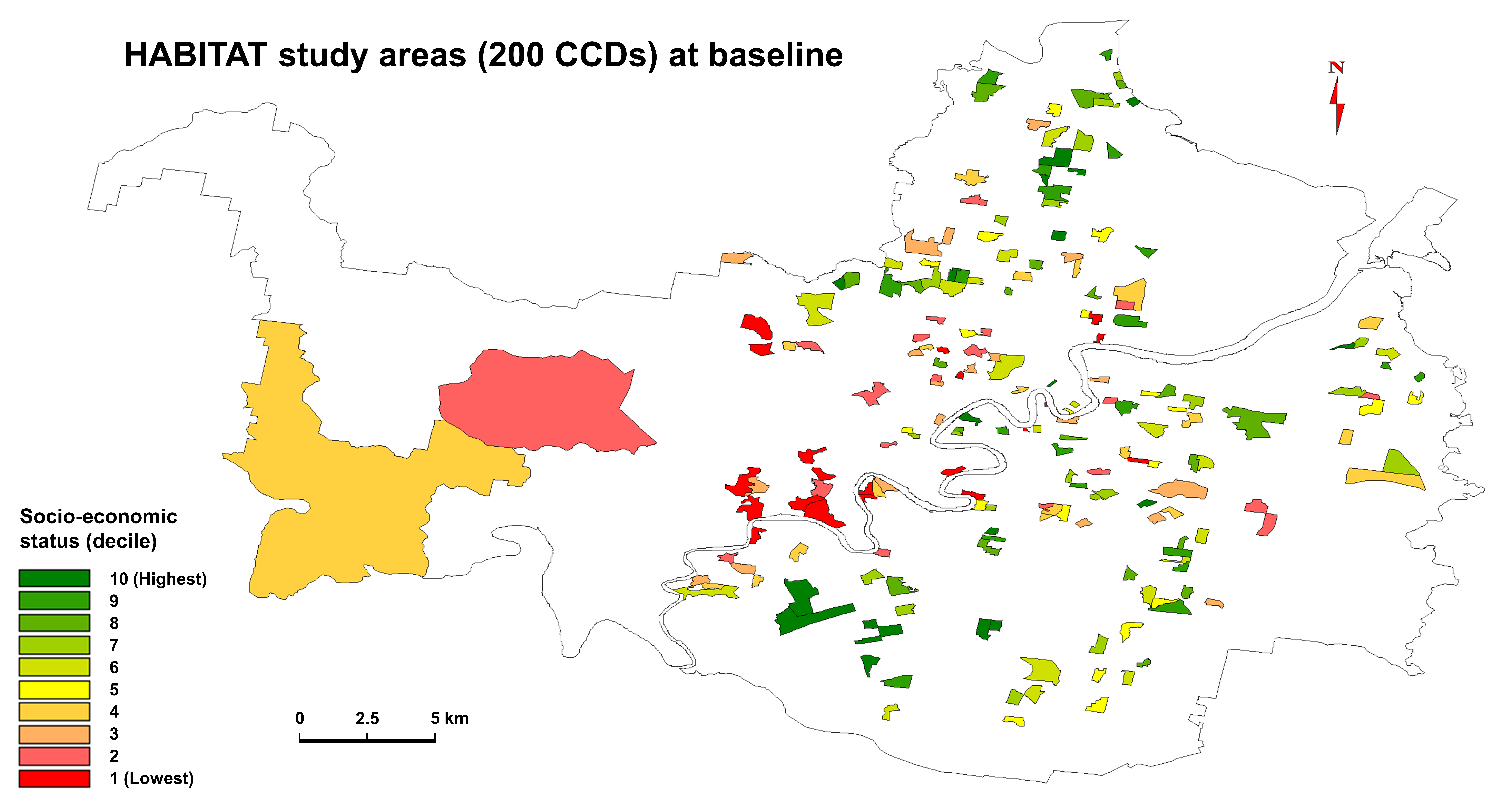

Supplement: S1 Fig — (TIF) [file pone.0252017.s001.tif]
