# Supplementary material for: Neighborhood environmental attributes and walking mobility decline: A longitudinal ecological study of mid-to-older aged Australian adults
Source: PLoS One. 2021 Jun 3;16(6):e0252017. doi: 10.1371/journal.pone.0252017 (PMC8174704; doi:10.1371/journal.pone.0252017)
Supplement: S1 Table — (DOCX) [file pone.0252017.s002.docx]

S1 Table. Ecological associations of walking mobility score change with environmental attributes at the neighborhood level

|  | Neighborhood level (N=195^a^) | | Neighborhood level (N=107^b^) | |
| --- | --- | --- | --- | --- |
|  | SVMs | MVM | SVMs | MVM |
| Residential density | -0.20 (-0.88, 0.48) | -0.14 (-0.88, 0.60) | 0.22 (-0.56, 0.99) | 0.38 (-0.58, 1.34) |
| Intersection density | -0.26 (-0.92, 0.39) | 0.31 (-0.41, 1.02) | -0.10 (-0.79, 0.59) | 0.09 (-0.73, 0.91) |
| Land use mix | 0.68 (0.03, 1.32)* | 0.56 (-0.18, 1.30) | 0.99 (0.34, 1.63)** | 0.87 (0.07, 1.66)* |
| Density of walking/bike paths | 0.57 (-0.08, 1.22)† | 0.80 (0.11, 1.50)* | -0.25 (-0.95, 0.45) | -0.38 (-1.12, 0.35) |
| Density of parks | 0.36 (-0.29, 1.00) | -0.16 (-0.90, 0.58) | 0.73 (0.09, 1.38)* | 0.28 (-0.48, 1.05) |
| Density of bus stops | -0.46 (-1.13, 0.21) | -0.34 (-1.05, 0.38) | -0.21 (-0.88, 0.45) | 0.21 (-0.52, 0.94) |
| Density of social incivilities | -1.02 (-1.65, -0.39)** | -1.08 (-1.81, -0.35)** | -0.53 (-1.19, 0.13) | -0.48 (-1.18, 0.22) |

† p < 0.1, * p < 0.05, ** p < 0.01

Regression coefficients (corresponding to a 1-SD increment in each exposure measure) and 95% CI are shown.

^a^ Neighborhoods with less than 5 participants excluded; ^b^ Neighborhoods with less than 15 participants excluded

SVMs (single variable models) examined each environmental attribute separately (not adjusted for each other). MVM (multi-variable model) examined all environmental attributes simultaneously (adjusted for each other).

All models adjusted for mean age, the mean proportion of women, those with high school qualification or less, those working, those living as a couple, and those with a higher income (over $93,600 per annum), area size, mean preference for walk-friendly and safe area, and area-level socio-economic status.
